# Supplementary figures and images for: Disease-Relevant Single Cell Photonic Signatures Identify S100β Stem Cells and their Myogenic Progeny in Vascular Lesions
Source: Stem Cell Rev Rep. 2021 Mar 17;17(5):1713–40. doi: 10.1007/s12015-021-10125-x (PMC8446106; doi:10.1007/s12015-021-10125-x)

A.

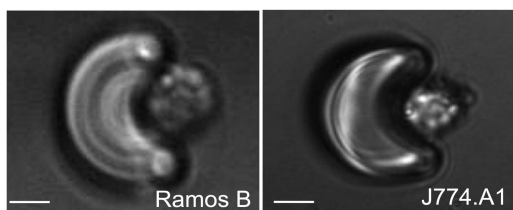

B.

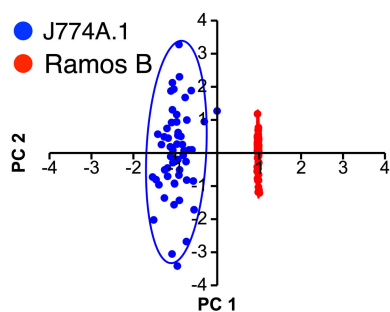

C.

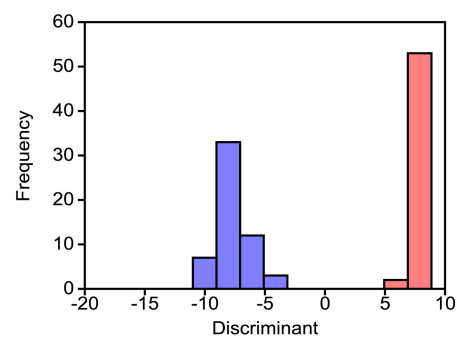

D.

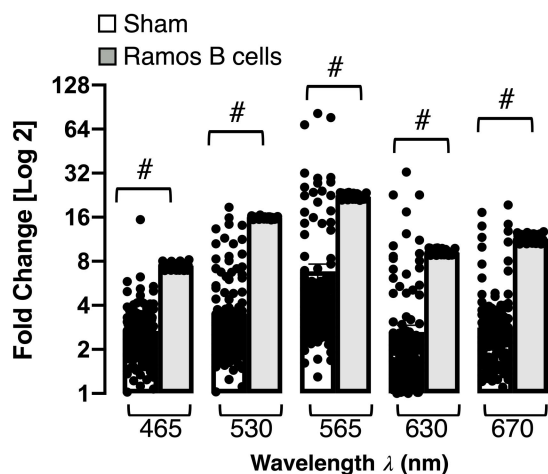

E.

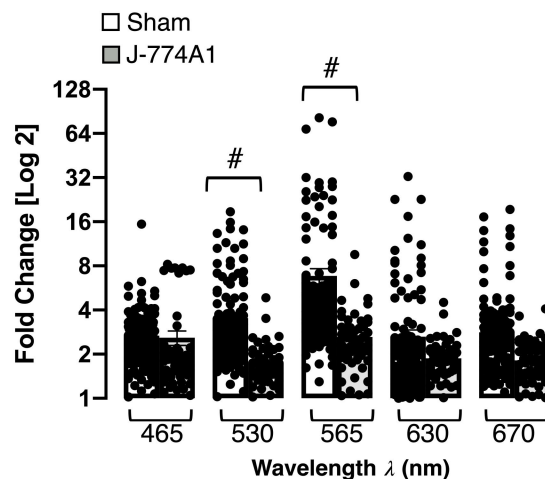

F.

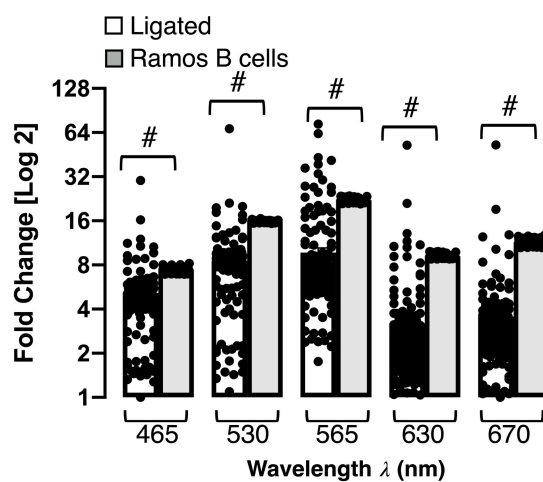

G.

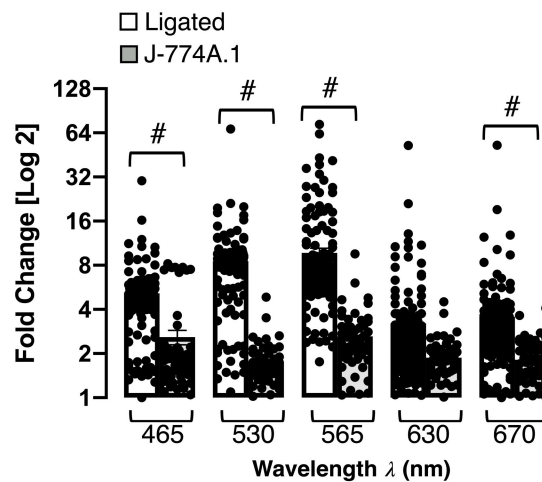

H.

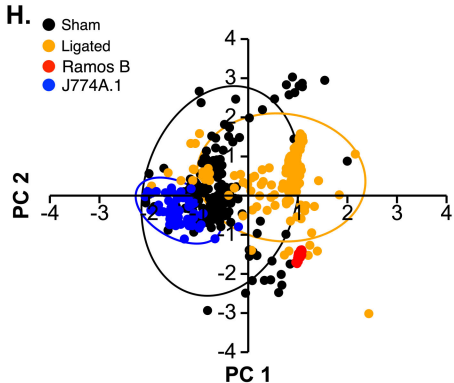

I.

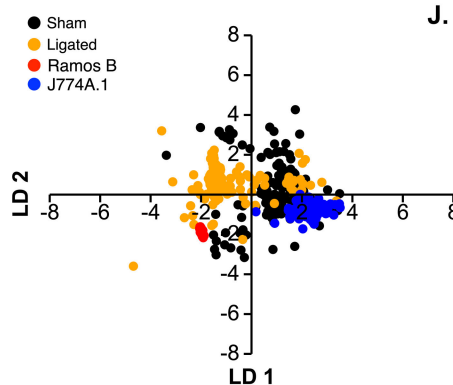

J.

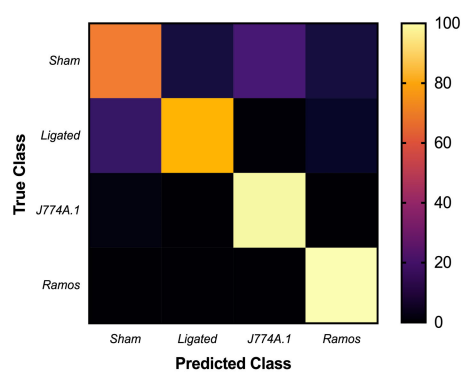

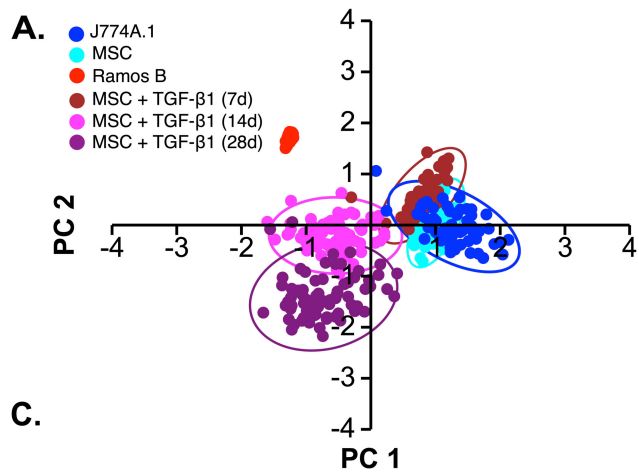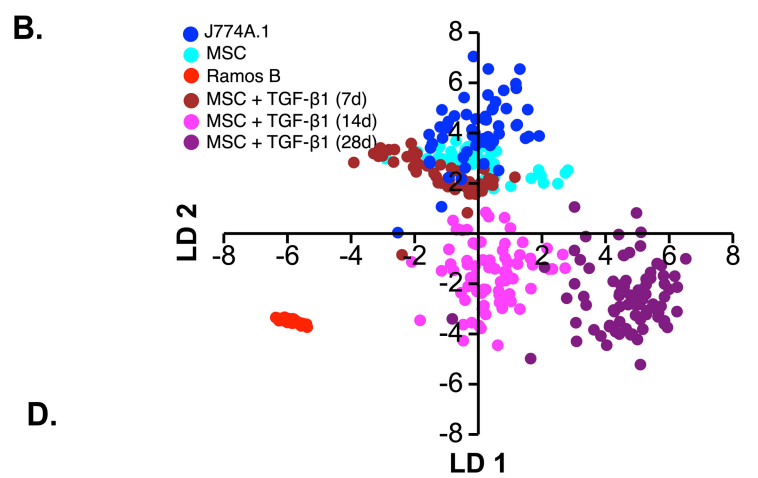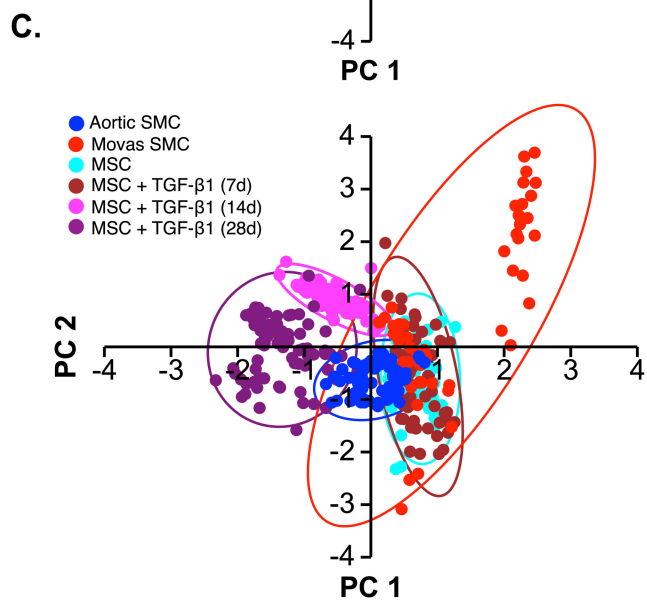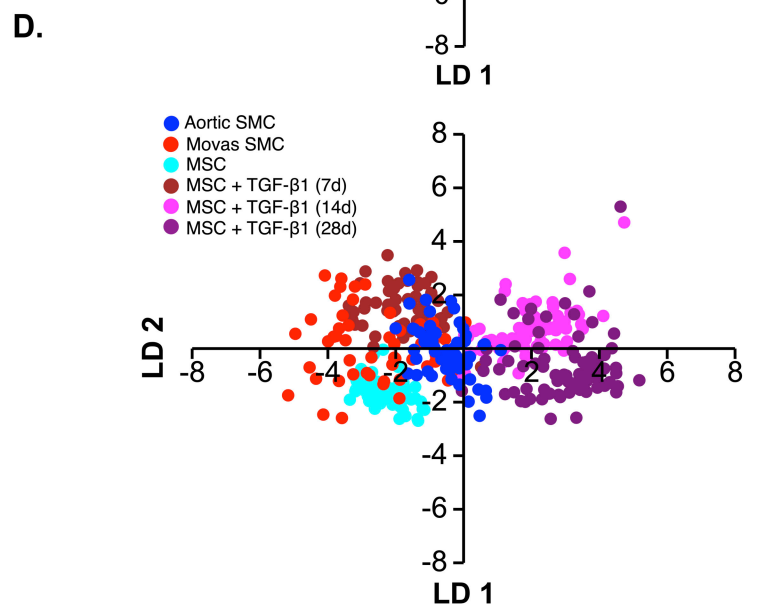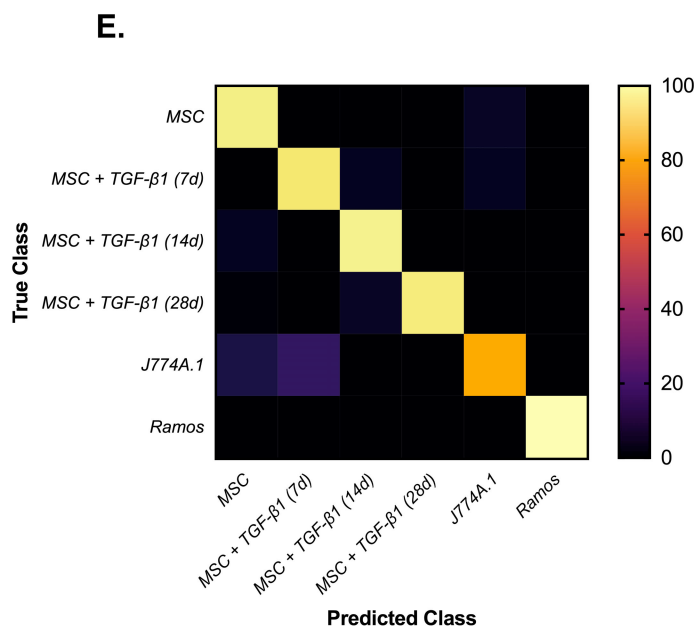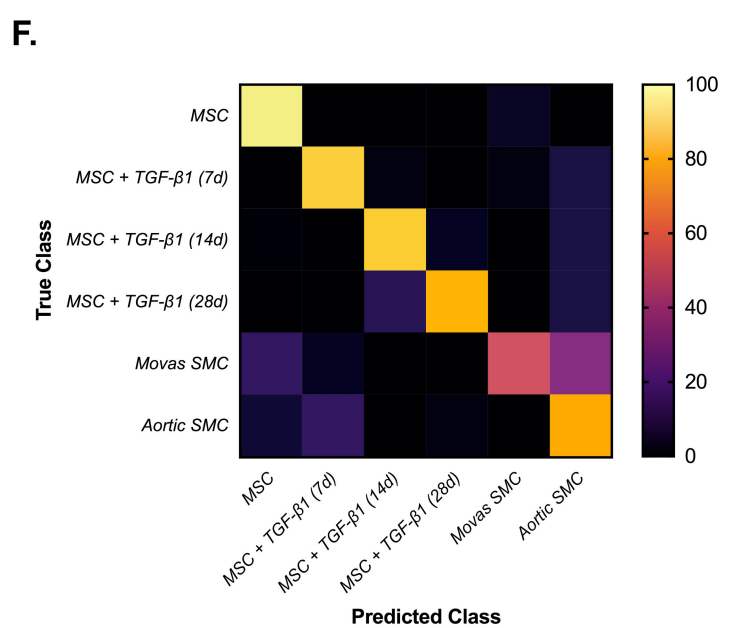

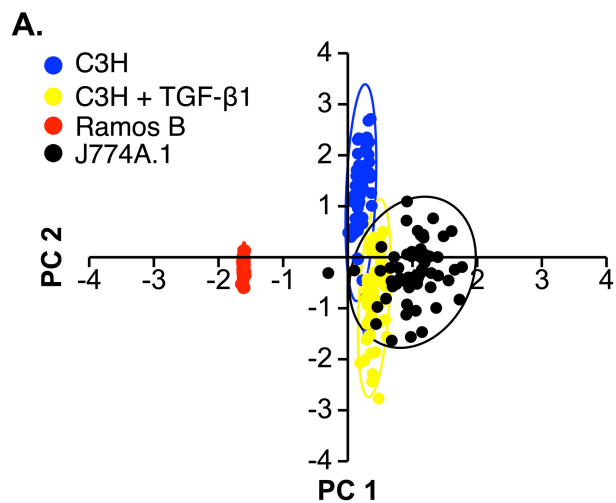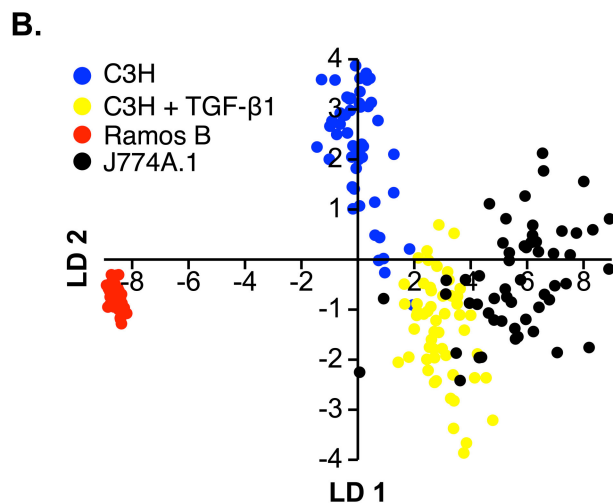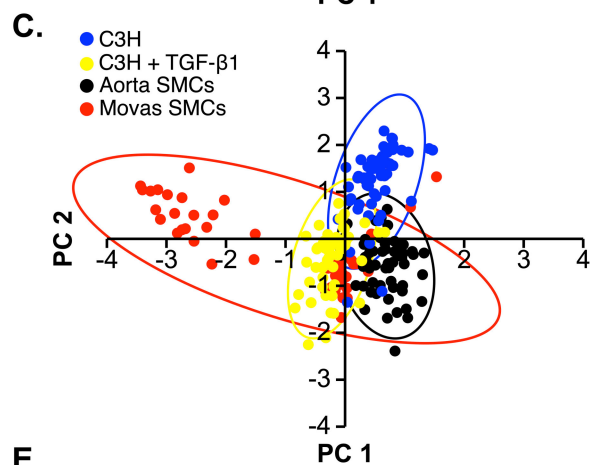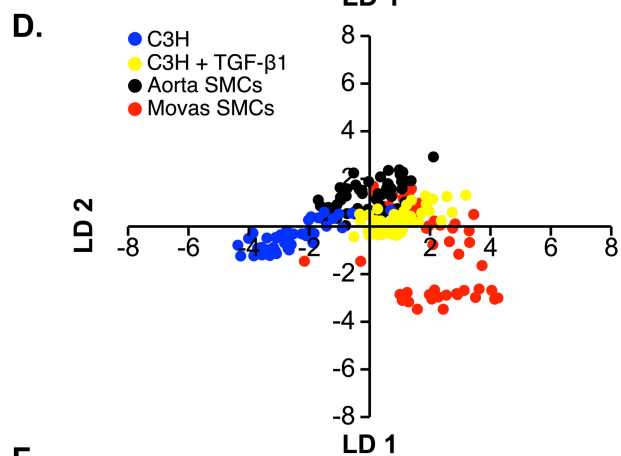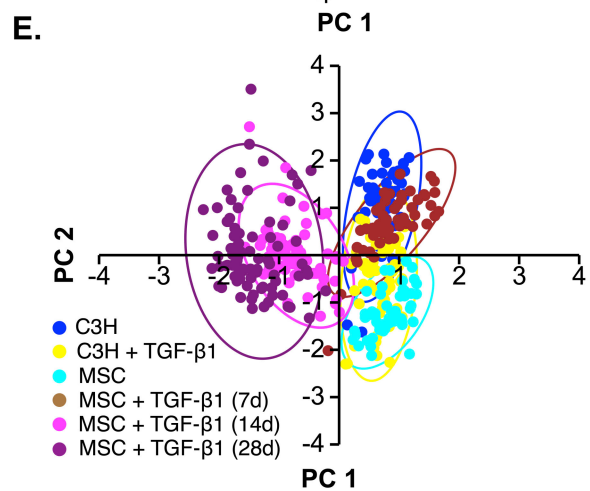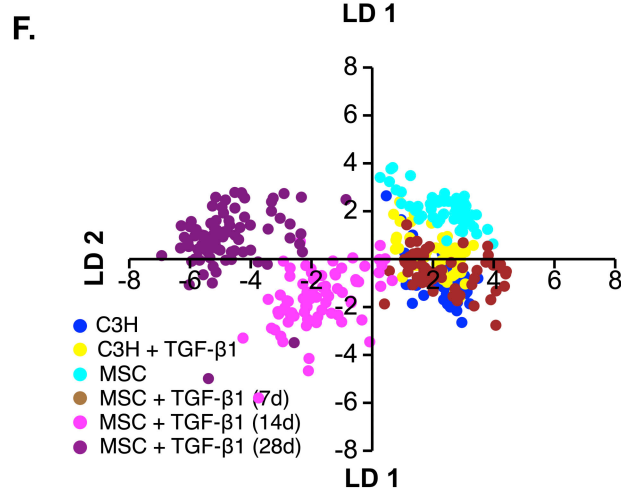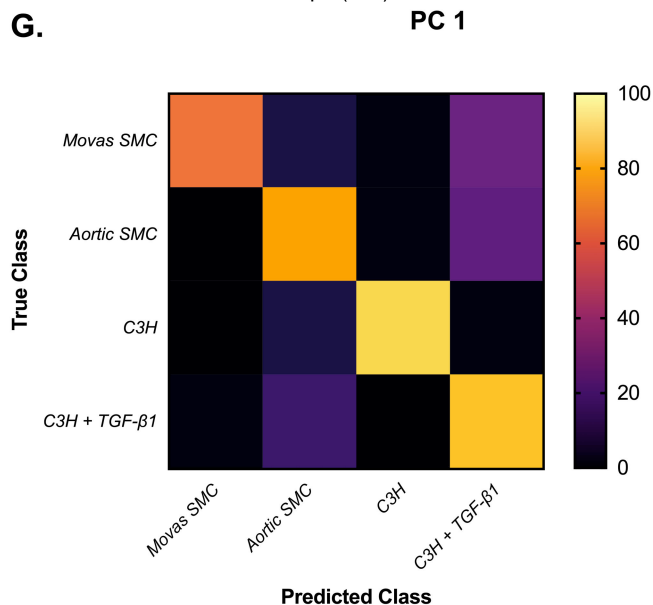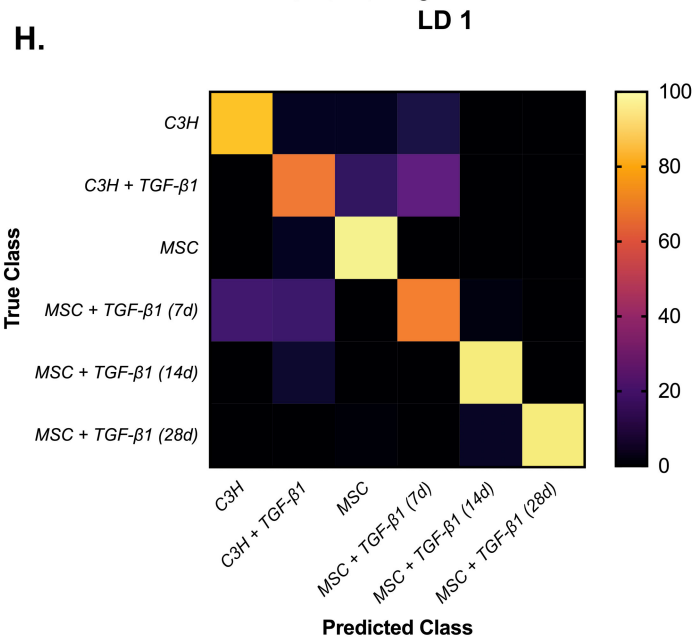

A.

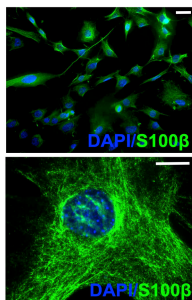

B.

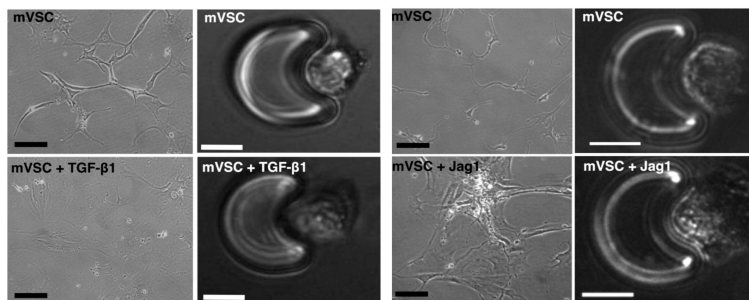

C.

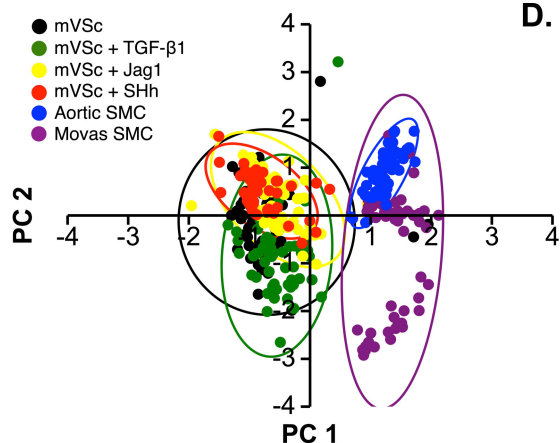

D.

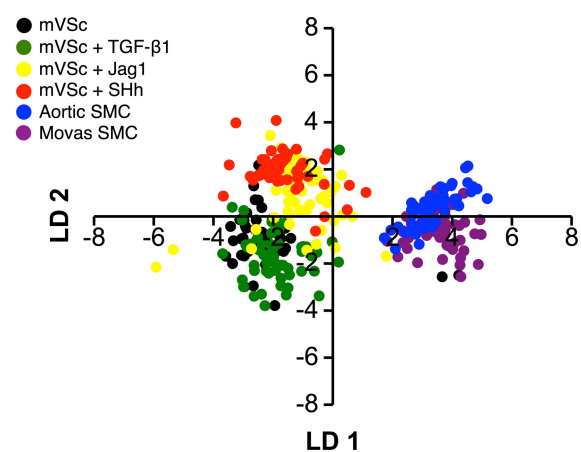

E.

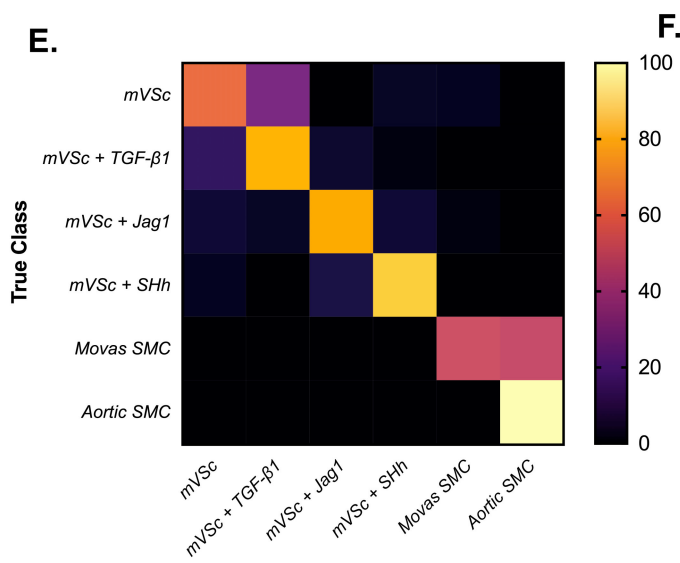

F.

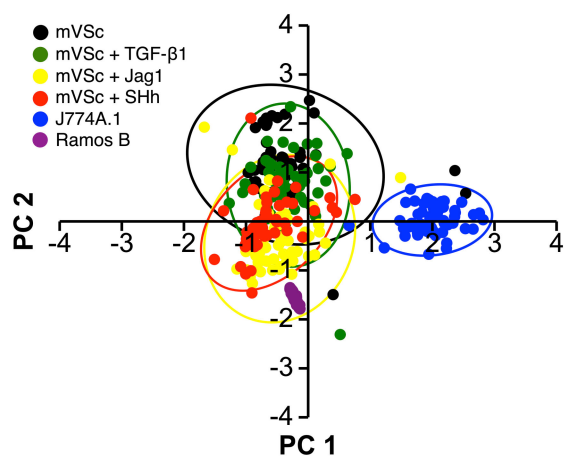

G.

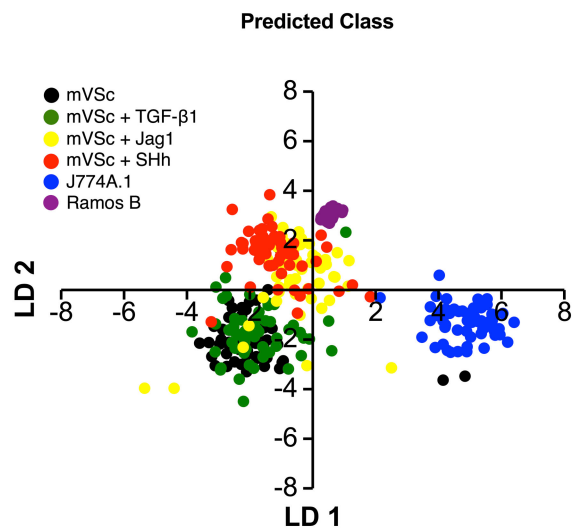

H.

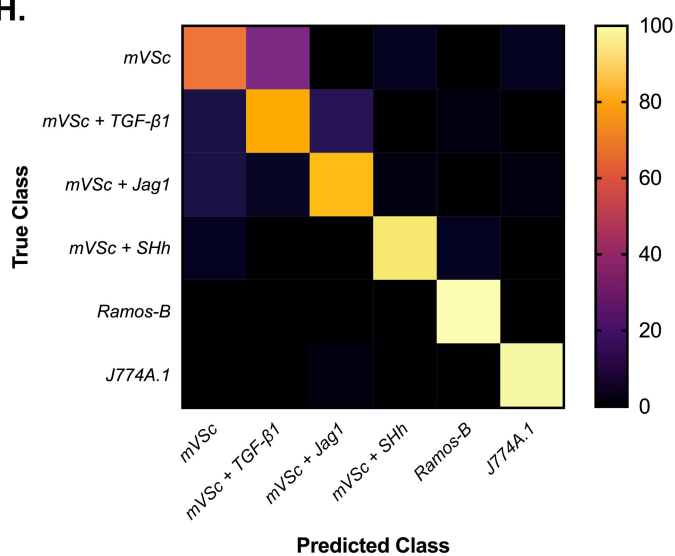

**A.**

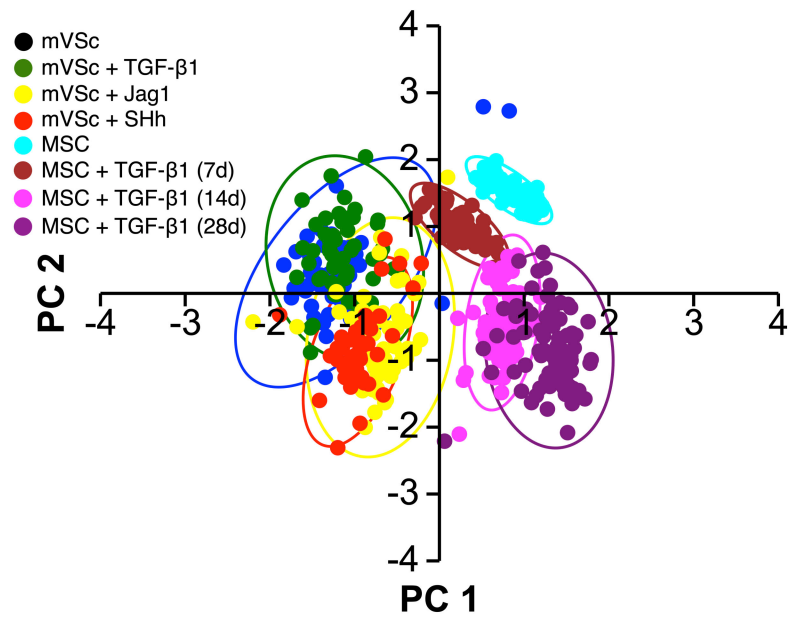

**B.**

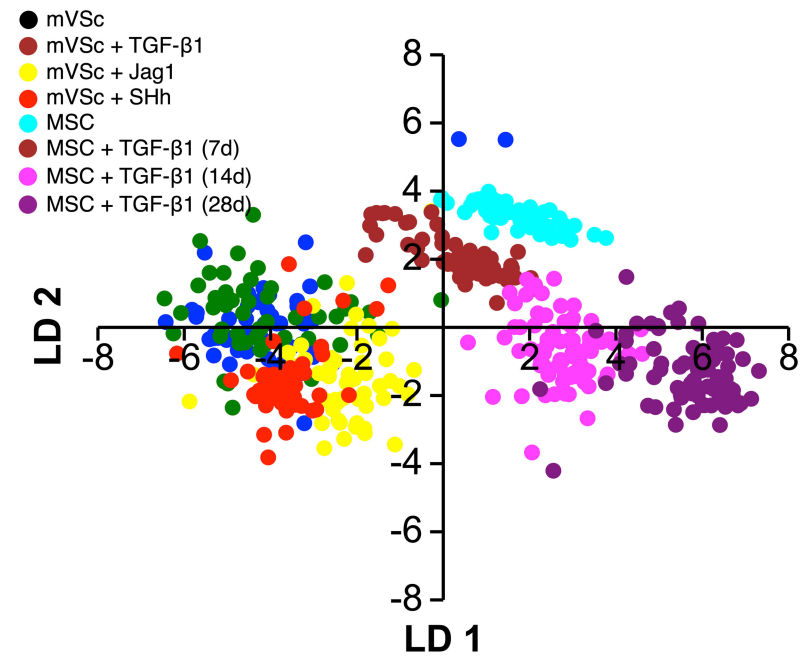

**C.**

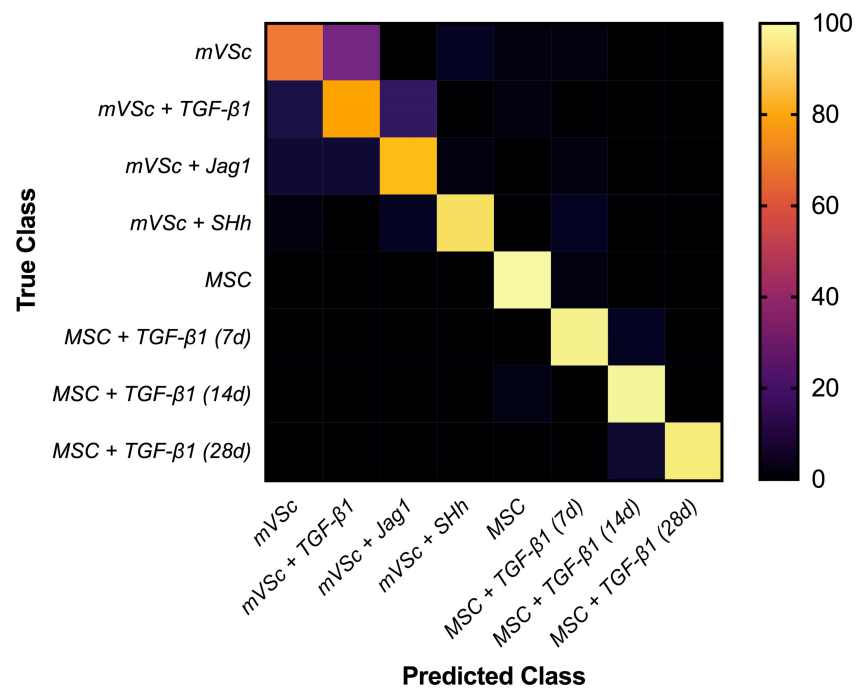

A.

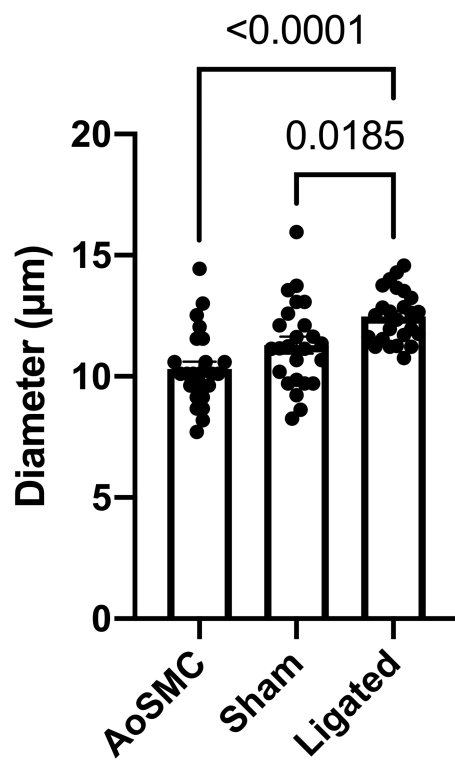

B.

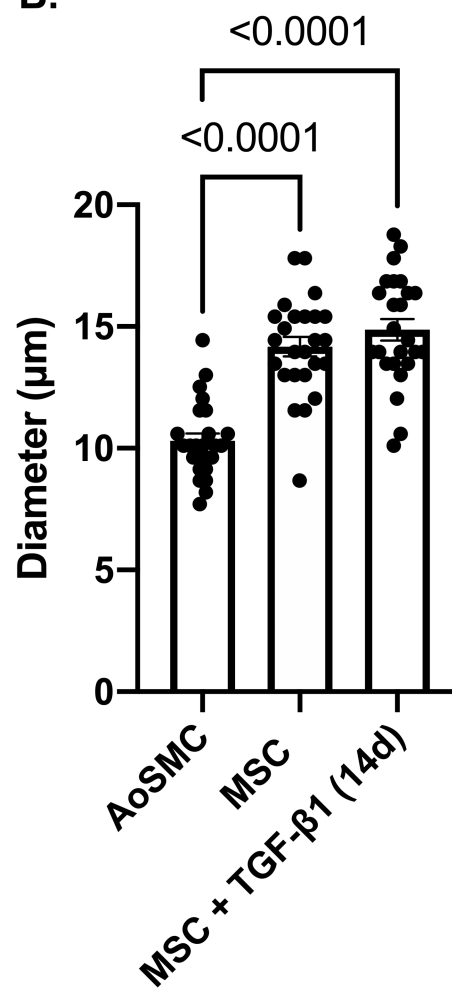

C.

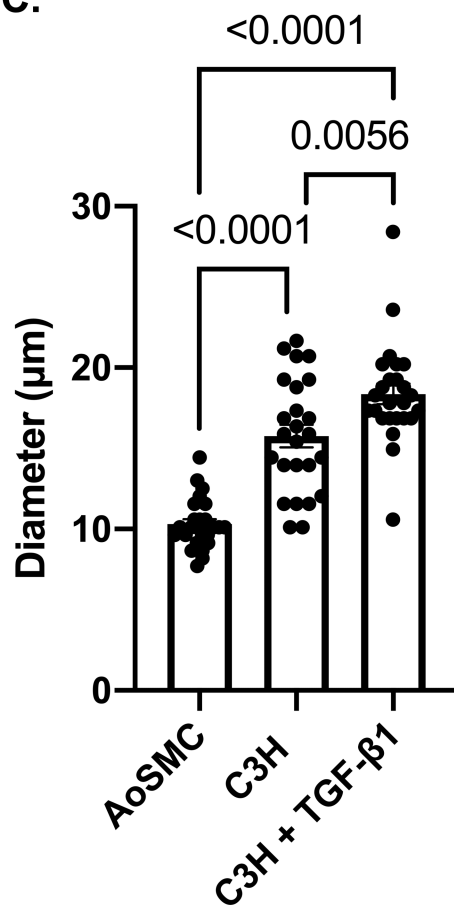

D.

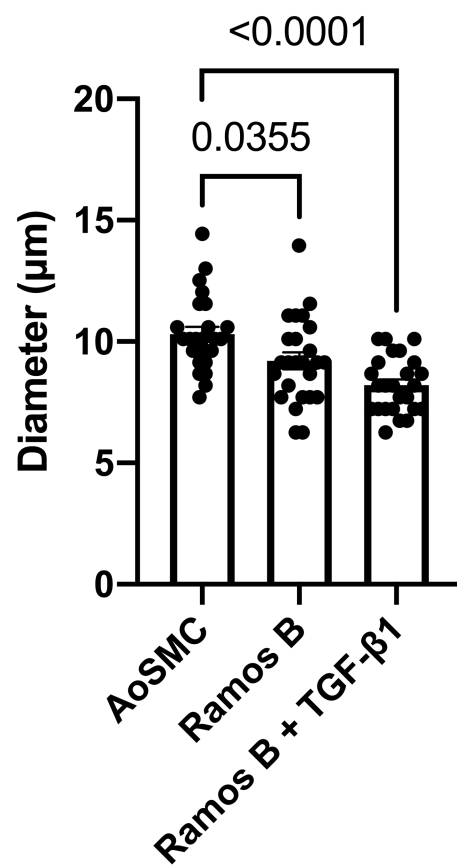

A.

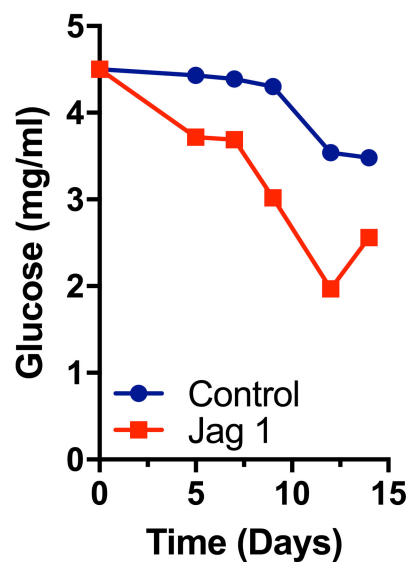

B.

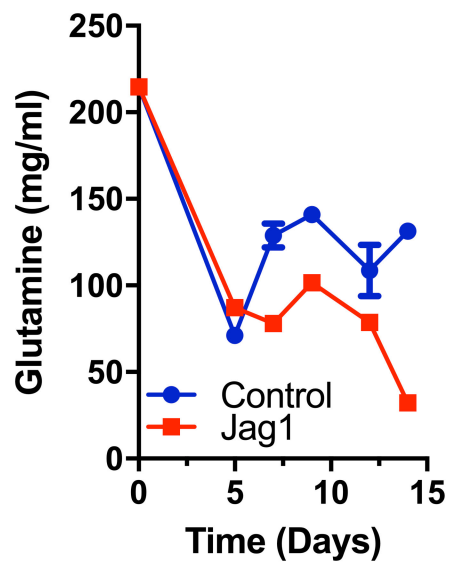

C.

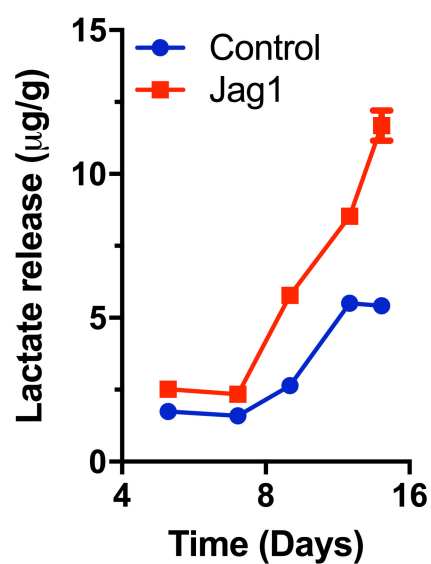

D.

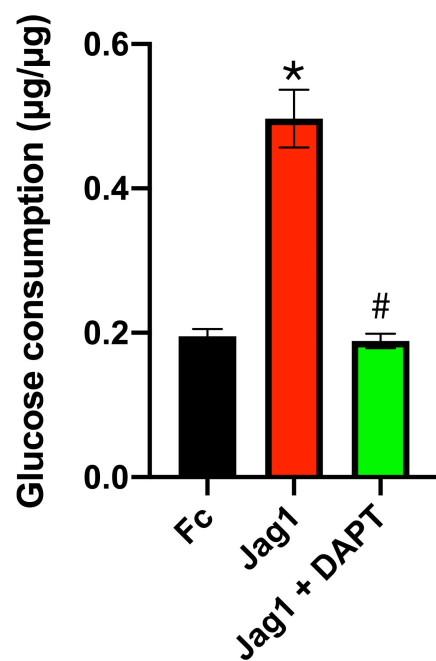

E.

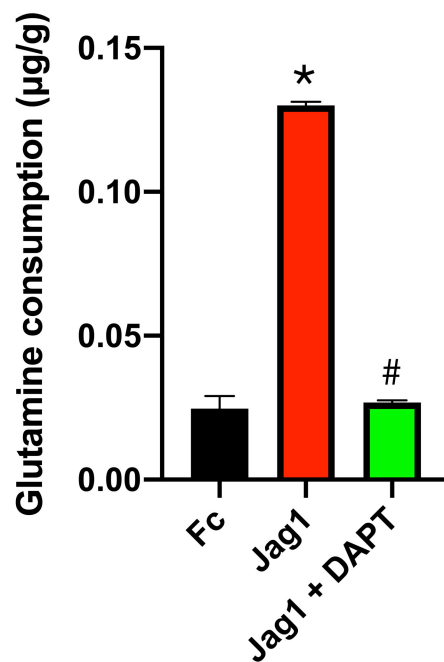

F.

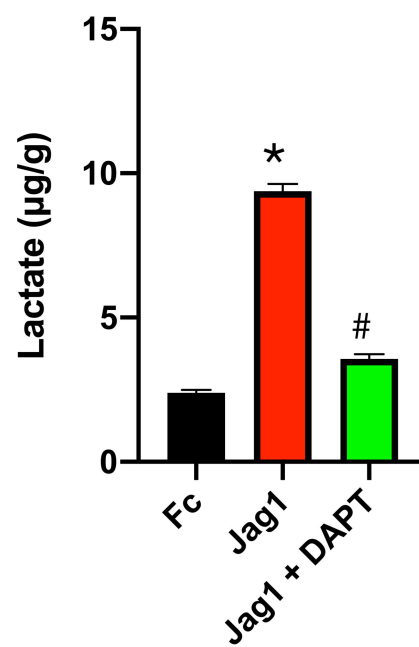

**A**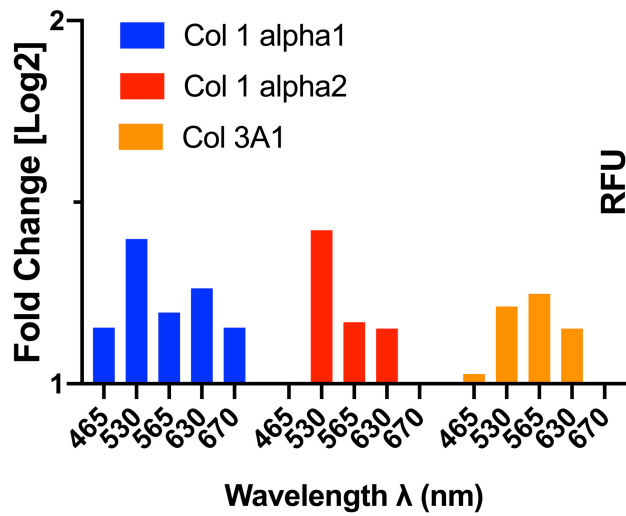**B**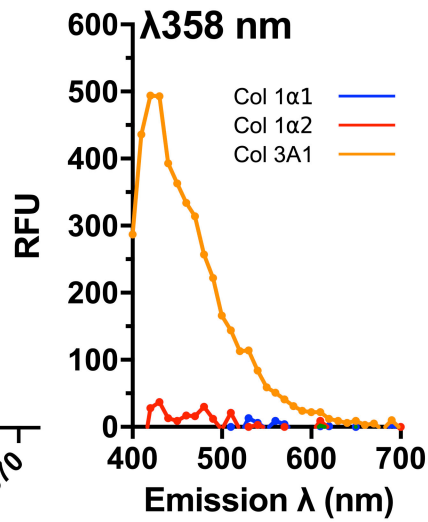**C**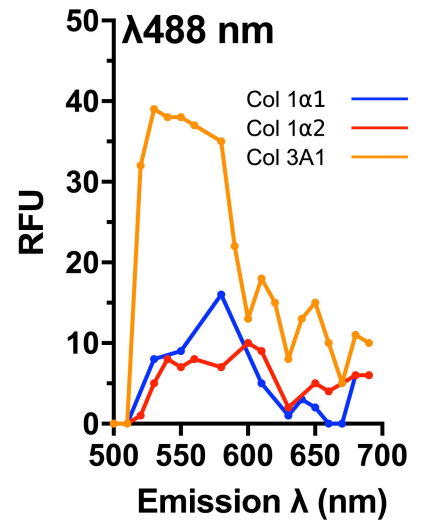**D**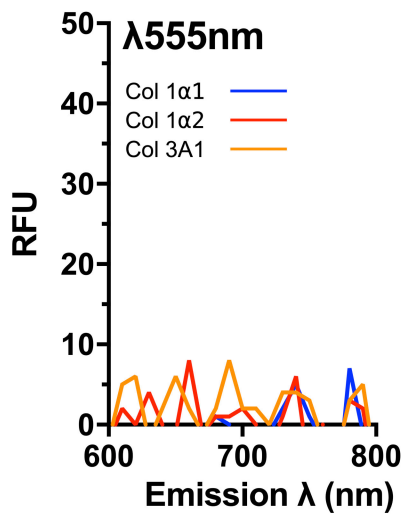**E**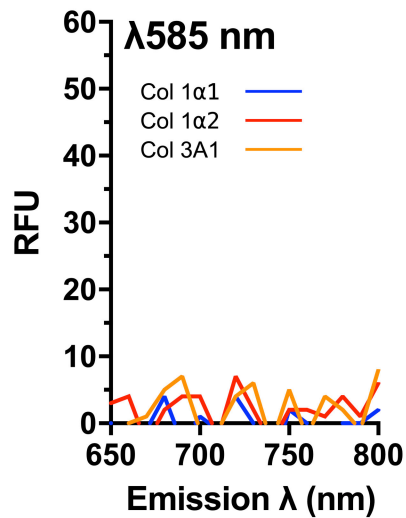**F**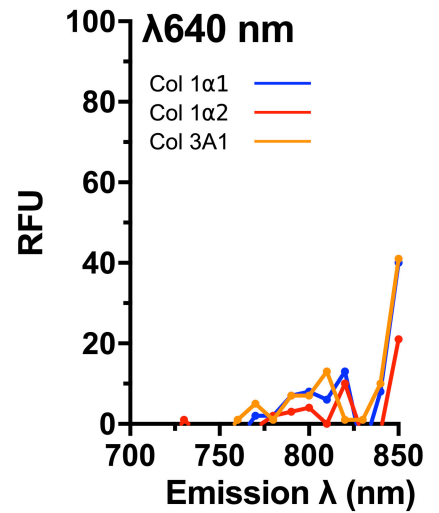**G**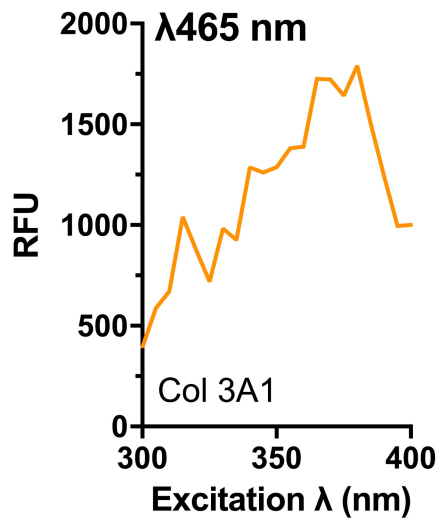**H**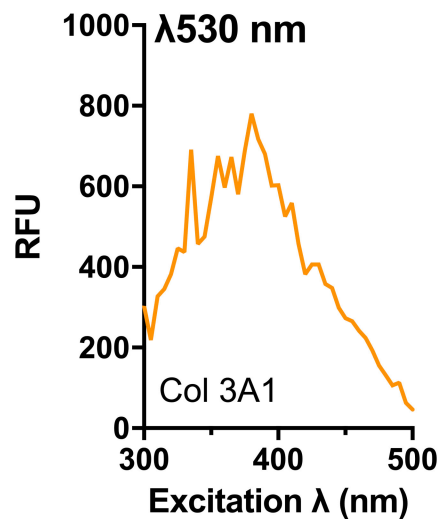**I**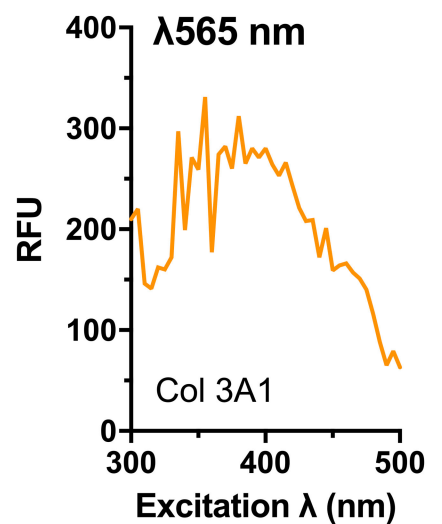

Supplement: Supplementary file 1 — (PDF 11430 kb) [file 12015_2021_10125_MOESM1_ESM.pdf]
