# Supplementary material for: Disease-Relevant Single Cell Photonic Signatures Identify S100β Stem Cells and their Myogenic Progeny in Vascular Lesions
Source: Stem Cell Rev Rep. 2021 Mar 17;17(5):1713–40. doi: 10.1007/s12015-021-10125-x (PMC8446106; doi:10.1007/s12015-021-10125-x)
Supplement: Supplementary file 2 — (PDF 9.37 mb) [file 12015_2021_10125_MOESM2_ESM.pdf]

Supplementary Table I

Antibodies and their corresponding dilutions used in immunohistochemistry.

Antibody/Product Name	Supplier/Product Number	Dilutions
Rabbit anti-mouse/rat alpha smooth muscle cell α-actin antibody	Abcam (ab5694)	1/200
Anti-Actin, α-Smooth Muscle antibody, Mouse monoclonal	Sigma (A5228)	1/200
Anti-S100-β (CT) Antibody, clone EP1576Y, rabbit monoclonal	Millipore (04-1054)	1/100
Chicken anti-GFP antibody	Abcam (ab13970)	1/1000
Rabbit Anti-RFP/dT antibody	Abcam (ab62341)	1/1000

Supplementary Table II

Antibodies and their corresponding dilutions used in immunocytochemistry/western blot.

Antibody/Product Name	Supplier/Product Number	Dilutions
Rabbit anti-mouse Calponin [EP798Y]	Abcam (ab46794)	1/200
Goat anti-mouse/rat/human smooth muscle Myosin heavy chain	Santa Cruz (sc-79079)	1/200
Rabbit Anti-S100β	Merck Millipore (ABN59)	1/100
Rabbit Anti-mouse/rat S100β [EP1576Y]	Abcam (ab52642)	1/100
Alexa Fluor® 488 Goat anti-mouse IgG	Invitrogen (A-11001)	1/1000
Alexa Fluor® 488 Goat anti-rabbit IgG	Invitrogen (A-11008)	1/1000

Alexa Fluor® 488 Donkey anti-goat IgG	Invitrogen (A-11055)	1/1000

Supplementary Table III

Antibodies used in Chromatin Immunoprecipitation (ChIP)

Antibody/Product Name	Supplier/Product Number
Rabbit anti-mouse Tri-Methyl-Histone H3 (Lys27) [C36B11]	Cell Signalling Technology (9733S)
Rabbit anti-mouse Di-Methyl-Histone H3 (Lys4) [C64G9]	Cell Signalling Technology (9725S)
Normal Rabbit IgG (ChIP graded)	Cell Signalling Technology (2729)

Supplementary Table IV

Customised primers used in this study from Integrity DNA Technology (IDT).

Customised primer		Sequences
Mm_Sm-mhc (Myh11)	Forward	5' - GCA GTG AGC TCT CAG TCA TC - 3'
	Reverse	5' - CAA TGC CTC CTC TGA CAA GT - 3'
Mm_Cnn1	Forward	5' - GCT TGT CTG CTG AAG TAA AGA AC - 3'
	Reverse	5' - TCC ATG AAG TTG TTC CCG ATG - 3'
Mm_Hprt	Forward	5' - GGC TAT AAG TTC TTT GCT GAC CTG C - 3'
	Reverse	5' - GCT TGC AAC CTT AAC CAT TTT GGG - 3'
Mm_Gapdh	Forward	5' - GCC TCC AAG GAG TAA GAA AC - 3'
	Reverse	5' - GCC TCC AAG GAG TAA GAA AC - 3'

Mm_ Sm-mhc (Myh11)	Forward	5’ - CCC TCC CTT TGC TAA ACA CA - 3'
for ChIP PCR	Reverse	5’ - CCA GAT CCT GGG TCC TTA CA - 3'

Primers used in this study from QIAGEN.

Primer	Product Name	Product Code
mHprt-1	Mm_Hprt_1_SG QuantiTect Primer Assay	QT00166768
mGapdh	Mm_Gapdh_3_SG QuantiTect Primer Assay	QT01658692
mCol3A1	Mm_Col3A1_3_SG QuantiTect Primer Assay	QT01055516

Supplemental Figure Legends

Figure S1. **Single cell photonics of Ramos B and J774A.1 macrophages. a.** Visualisation of Ramos B and J774A.1 cells on each V-cup in the LoaD platform. **b.** PCA loading plots of Ramos B and J774A.1 cells. **c.** LDA of Ramos B and J774A.1 cells. Data are from 55 cells/group over five wavelengths. **d-g.** Single cell auto-fluorescence photon emissions across five wavelengths from (D, F) Ramos B cells and (E, G) J774A.1 cells *in vitro* compared to sham (D, E) and ligated cells (F, G) *ex vivo*. Data are the Log2 fold increase and represent the mean ± SEM of 55-178 cells/group, #p≤0.001 vs Sham (D,E) Ligated (F,G). **h**. PCA loading plots of sham (black), ligated (orange), Ramos B (red) and J774A.1 (blue) cells. **i**. LDA plots of sham (black), ligated (orange) cells ex vivo and Ramos B (red) and J774A.1 (blue) cells in vitro. Data are from 466 cells across the five wavelengths **j**. Confusion matrix of true class and predicted class following a leave-one-out cross-validation procedure by the LDA classifier.

Figure S2. **Single cell photonics of MSC cells and their myogenic progeny compared to aortic SMC, Movas SMC, Ramos B cells and J774A.1 macrophages *in vitro*. a.** PCA loading plots of MSCs and their myogenic progeny, Ramos B cells and J774A.1 macrophages. Data are from 55-79 cells/group. **b.** LDA plot of MSCs and their myogenic progeny, Ramos B cells and J774A.1 macrophages. **c.** PCA loading plots of MSCs and their myogenic progeny, aortic SMCs and Movas SMCs. **d.** LDA plots of MSCs and their myogenic progeny, aortic SMCs and Movas SMCs. Data are from 55-79 cells/group. **e.** Confusion matrix of true class and predicted class following a leave-one-out cross-validation procedure using the LDA classifier. Data are from 378 cells across five wavelengths. **f.** Confusion matrix of true class and predicted class following a leave-one-out cross-validation procedure using the LDA classifier. Data are from 378 cells across five wavelengths.

Figure S3. **Single cell photonics of C3H10T1/2 cells and their myogenic progeny compared to Ramos B, J774A.1, aortic SMC, Movas SMC and MSC and their myogenic progeny *in vitro*. a.** PCA loading plots of C3H10T1/2 cells and their myogenic progeny compared to Ramos B cells and J774A1 cells. **b.** LDA plots of C3H10T1/2 cells and their myogenic progeny compared to Ramos B cells and J774A1 **c.** PCA loading plots of C3H10T1/2 cells and their myogenic progeny, aortic SMCs and Movas SMCs. Data are from 55 cells/group. **d.** LDA plots of C3H10T1/2 cells and their myogenic progeny, aortic SMCs and Movas SMCs. **e.** PCA loading plots of C3H10T1/2 cells and their myogenic progeny, MSCs and their myogenic progeny. Data are from 55-79 cells/group. **f.** LDA plots of C3H10T1/2 cells and their myogenic progeny, MSCs and their myogenic progeny. **g.** Confusion matrix of true class and predicted class following a leave-one-out cross-validation procedure using the LDA classifier. Data are from 220 cells across five wavelengths. **h.** Confusion matrix of true class and predicted class following a leave-one-out cross-validation procedure using the LDA classifier. Data are from 367 cells across five wavelengths.

Figure S4. **Single cell photonics of mVSc and their myogenic progeny compared to aortic SMC, Movas SMC, Ramos B and J774A.1 macrophages.** **a.** Representative immunocytochemical analysis of S100β expression in mVSc. **b.** Visualisation of mVSc and their myogenic progeny on each V-cup in the LoaD platform. **c.** PCA loading plots of mVSc and their myogenic progeny, aortic SMCs and Movas SMCs. Data are from 55 cells/group. **d.** LDA plot of mVSc and their myogenic progeny, aortic SMCs and Movas SMCs. Data are from 55 cells/group. **e.** Confusion matrix of true class and predicted class following a leave-one-out cross-validation procedure using the LDA classifier. Data are from 319 cells across five wavelengths. **f.** PCA loading plots of mVSc and their myogenic progeny, Ramos B cells and J774A.1 macrophages. Data are from 55 cells/group. **g.** LDA plot of mVSc and their myogenic progeny, Ramos B cells and J774A.1 macrophages. Data are from 55 cells/group. **h.** Confusion matrix of true class and predicted class following a leave-one-out cross-validation procedure using the LDA classifier. Data are from 319 cells across five wavelengths.

Figure S5. **Single cell photonics of mVSc and their myogenic progeny compared to MSCs and their myogenic progeny.** **a.** PCA loading plots of mVSc and their myogenic progeny, and MSC and their myogenic progeny. Data are from 55-79 cells/group. **b.** LDA plot of mVSc and their myogenic progeny, and MSC and their myogenic progeny. Data are from 55-79 cells/group. **c.** Confusion matrix of true class and predicted class following a leave-one-out cross-validation procedure using the LDA classifier. Data are from 487 cells across five wavelengths.

Figure S6. **Single cell diameter measurements of cells isolated from sham and ligated vessels *ex vivo,* and aortic SMCs, Ramos B cells and myogenic progeny *in vitro*.** **a.** Diameter measurements of cells isolated from sham and ligated vessels captured on V-cups compared to aortic SMCs. **b.** Diameter measurements of MSCs before and after myogenic differentiation with TGF-β1 for 14d. **c.** Diameter measurements of C3H 10T1/2 cells before and after myogenic differentiation with TGF-β1 for 14 d. **d.** Diameter measurements of Ramos B cells before and after treatment with TGF-β1 for 14 d. Data are from 25 cells/group.

Figure S7. **The effect of myogenic differentiation on S100β stem cell metabolism.** **a.** The level of glucose, **b.** glutamine and **c.** lactate release in conditioned media from S100β mVSc before and after treatment with the myogenic stimulus, Jag-1 (1μg/ml) over time. **d-f.** The level of glucose consumption, glutamine consumption and lactate release in conditioned media after treatment with Jag-1 (1μ/ml) in the absence or presence of Notch inhibitor , DAPT (100μM). *$p<0.05$ vs Fc Control, # $p<0.05$ vs Jag1. Data are mean ± SEM of three individual wells.

Figure S8. **Collagen autofluorescence.** **a**. Representative autofluorescence photon emissions from recombinant Col 1α1, Col 1α2, and Col3A1 across five wavelengths using the Load platform. Date are corrected for background emissions from media. Data are the Log2 fold increase and represent the mean of three samples **b-f.** Autofluorescence emission spectra from Col 1α1, Col 1α2, and Col3A1 following excitation at λ358- 565 nm. Data are representative of two independent experiments. **g-i.** Autofluorescence emission spectra from

Col3A1 at λ465, λ530 and λ565 following excitation at λ300- 500 nm. Data are representative of two independent experiments. Date are corrected for background emissions from PBS.

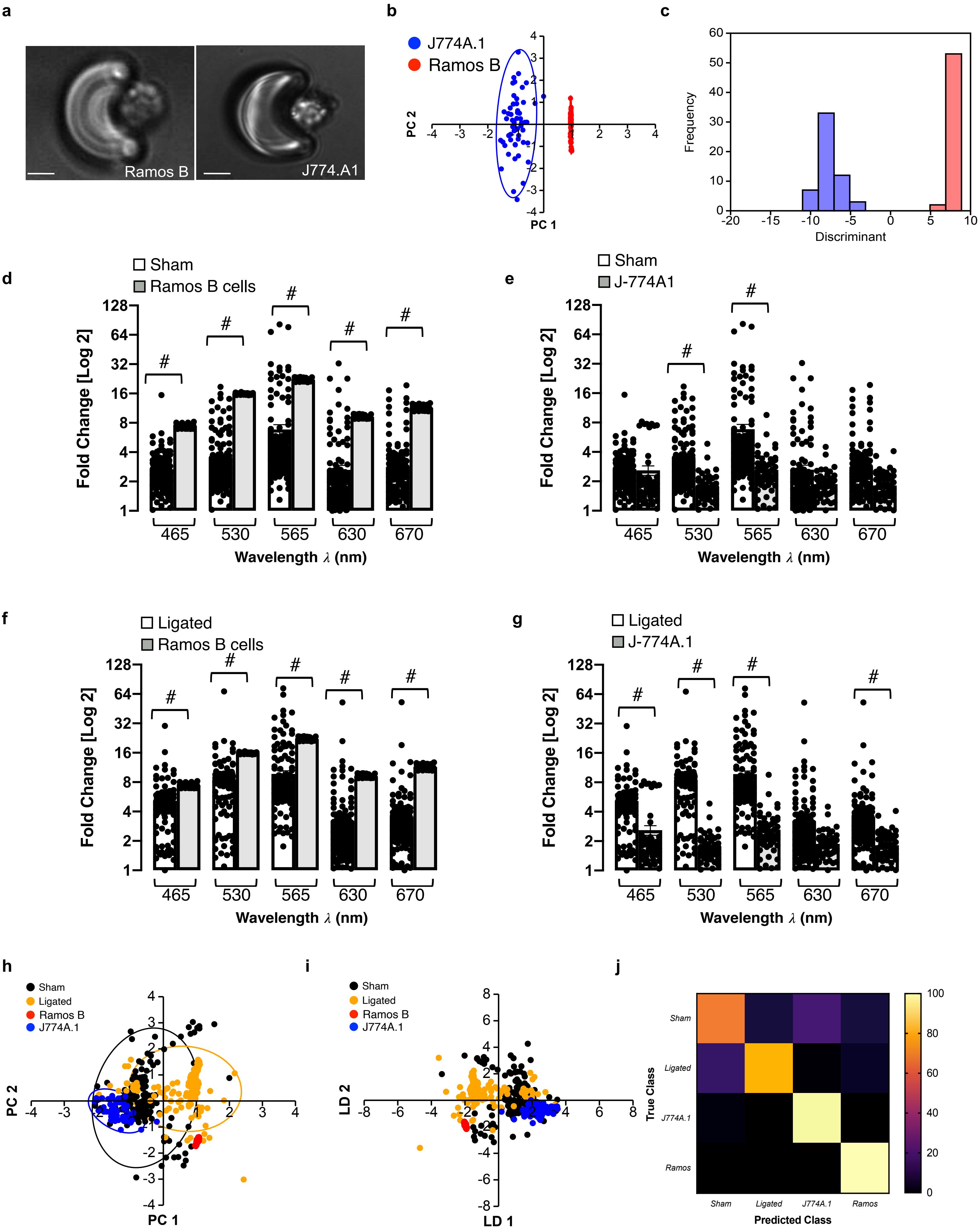

a
Ramos B
J774.A1
b
J774A.1
Ramos B
PC 2
PC 1
c
Frequency
Discriminant
d
Sham
Ramos B cells
Fold Change [Log 2]
Wavelength λ (nm)
e
Sham
J-774A1
Fold Change [Log 2]
Wavelength λ (nm)
f
Ligated
Ramos B cells
Fold Change [Log 2]
Wavelength λ (nm)
g
Ligated
J-774A.1
Fold Change [Log 2]
Wavelength λ (nm)
h
Sham
Ligated
Ramos B
J774A.1
PC 2
PC 1
i
Sham
Ligated
Ramos B
J774A.1
LD 2
LD 1
j
True Class
Sham
Ligated
J774A.1
Ramos
Predicted Class

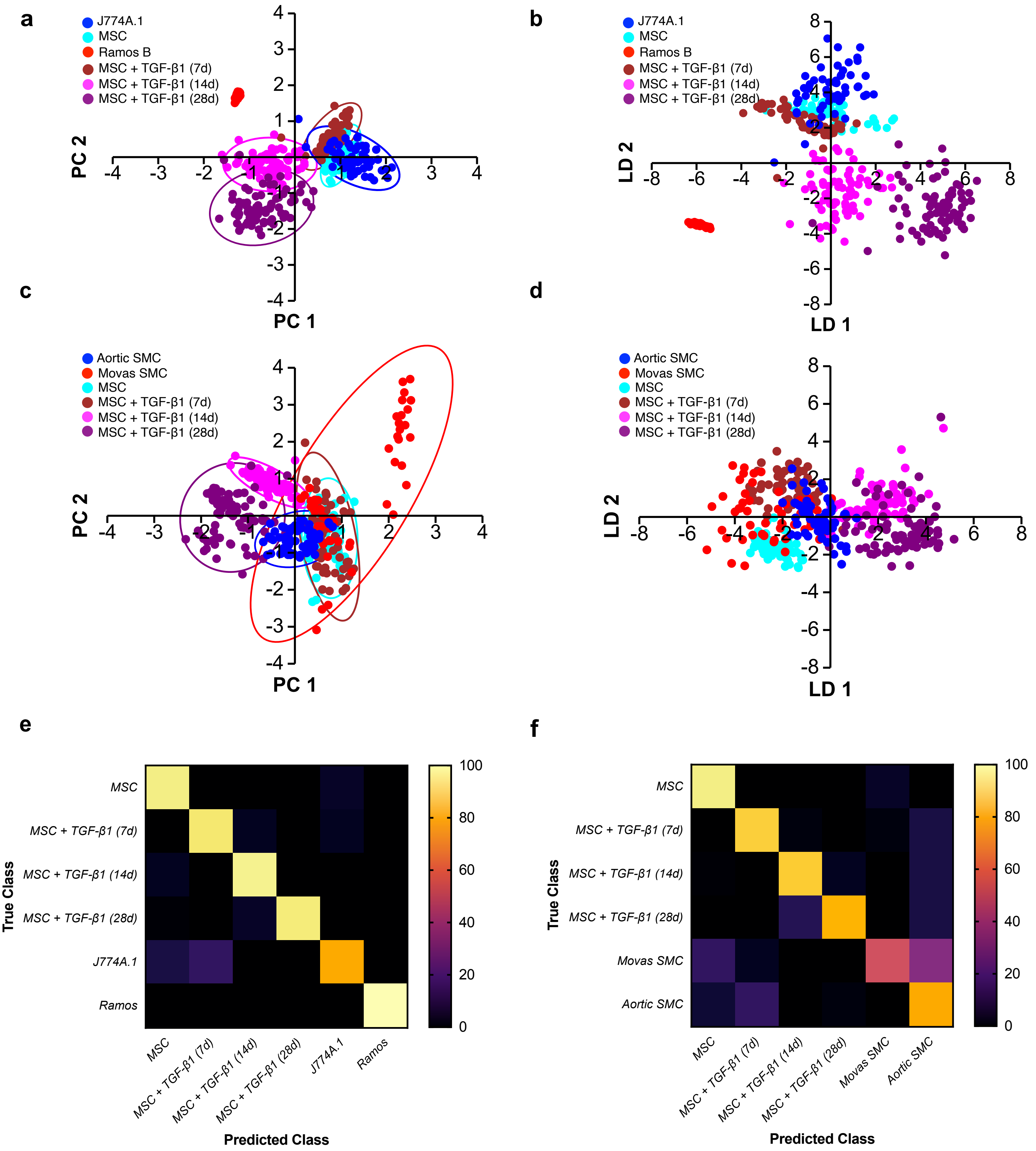
a
J774A.1
MSC
Ramos B
MSC + TGF-β1 (7d)
MSC + TGF-β1 (14d)
MSC + TGF-β1 (28d)
PC 2
PC 1
b
J774A.1
MSC
Ramos B
MSC + TGF-β1 (7d)
MSC + TGF-β1 (14d)
MSC + TGF-β1 (28d)
LD 2
LD 1
c
Aortic SMC
Movas SMC
MSC
MSC + TGF-β1 (7d)
MSC + TGF-β1 (14d)
MSC + TGF-β1 (28d)
PC 2
PC 1
d
Aortic SMC
Movas SMC
MSC
MSC + TGF-β1 (7d)
MSC + TGF-β1 (14d)
MSC + TGF-β1 (28d)
LD 2
LD 1
e
True Class
MSC
MSC + TGF-β1 (7d)
MSC + TGF-β1 (14d)
MSC + TGF-β1 (28d)
J774A.1
Ramos
Predicted Class
MSC
MSC + TGF-β1 (7d)
MSC + TGF-β1 (14d)
MSC + TGF-β1 (28d)
J774A.1
Ramos
100
80
60
40
20
0
f
True Class
MSC
MSC + TGF-β1 (7d)
MSC + TGF-β1 (14d)
MSC + TGF-β1 (28d)
Movas SMC
Aortic SMC
Predicted Class
MSC
MSC + TGF-β1 (7d)
MSC + TGF-β1 (14d)
MSC + TGF-β1 (28d)
Movas SMC
Aortic SMC
100
80
60
40
20
0

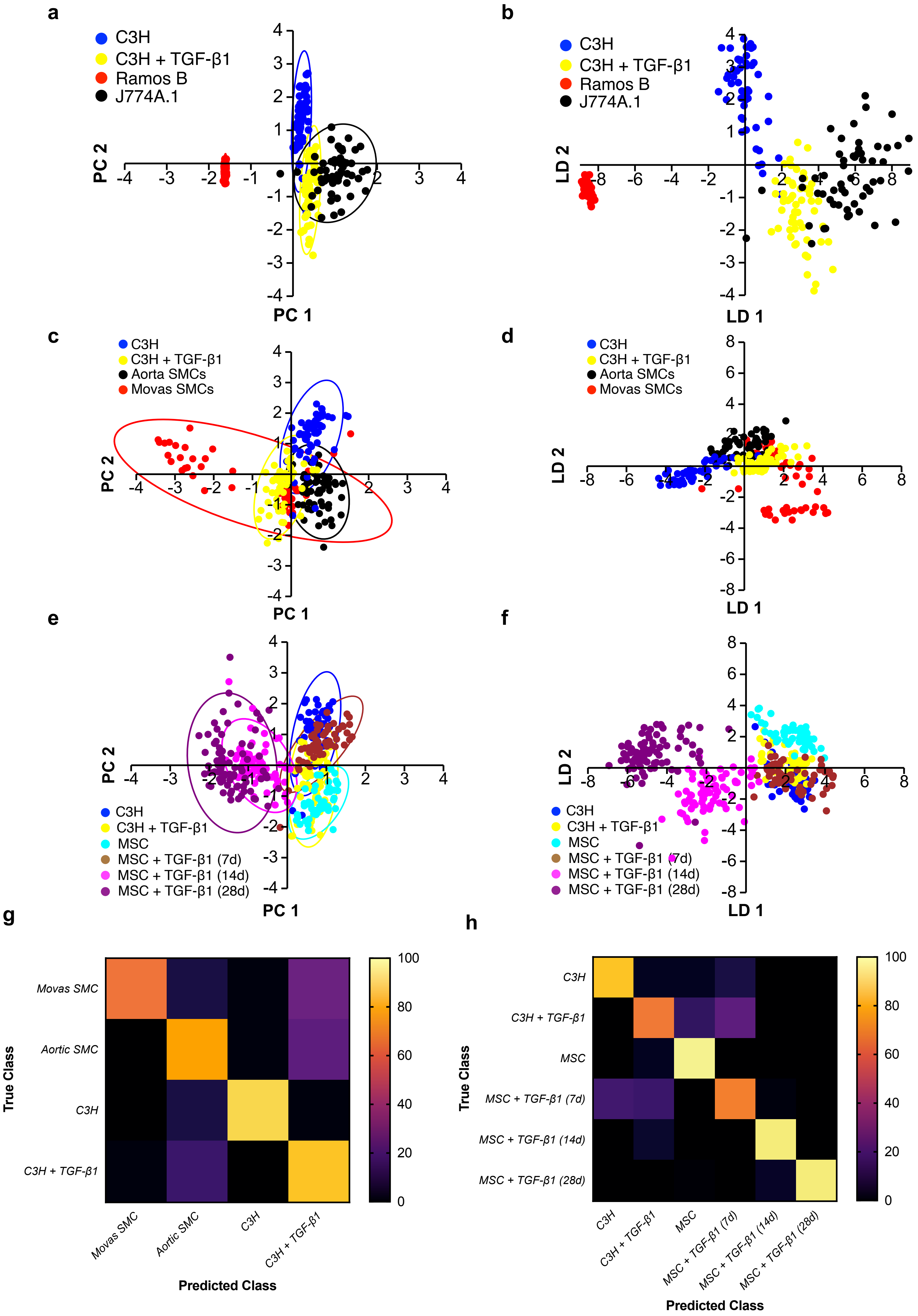
a
C3H
C3H + TGF-β1
Ramos B
J774A.1
PC 2
PC 1
b
C3H
C3H + TGF-β1
Ramos B
J774A.1
LD 2
LD 1
c
C3H
C3H + TGF-β1
Aorta SMCs
Movas SMCs
PC 2
PC 1
d
C3H
C3H + TGF-β1
Aorta SMCs
Movas SMCs
LD 2
LD 1
e
C3H
C3H + TGF-β1
MSC
MSC + TGF-β1 (7d)
MSC + TGF-β1 (14d)
MSC + TGF-β1 (28d)
PC 2
PC 1
f
C3H
C3H + TGF-β1
MSC
MSC + TGF-β1 (7d)
MSC + TGF-β1 (14d)
MSC + TGF-β1 (28d)
LD 2
LD 1
g
True Class
Movas SMC
Aortic SMC
C3H
C3H + TGF-β1
Predicted Class
h
True Class
C3H
C3H + TGF-β1
MSC
MSC + TGF-β1 (7d)
MSC + TGF-β1 (14d)
MSC + TGF-β1 (28d)
Predicted Class

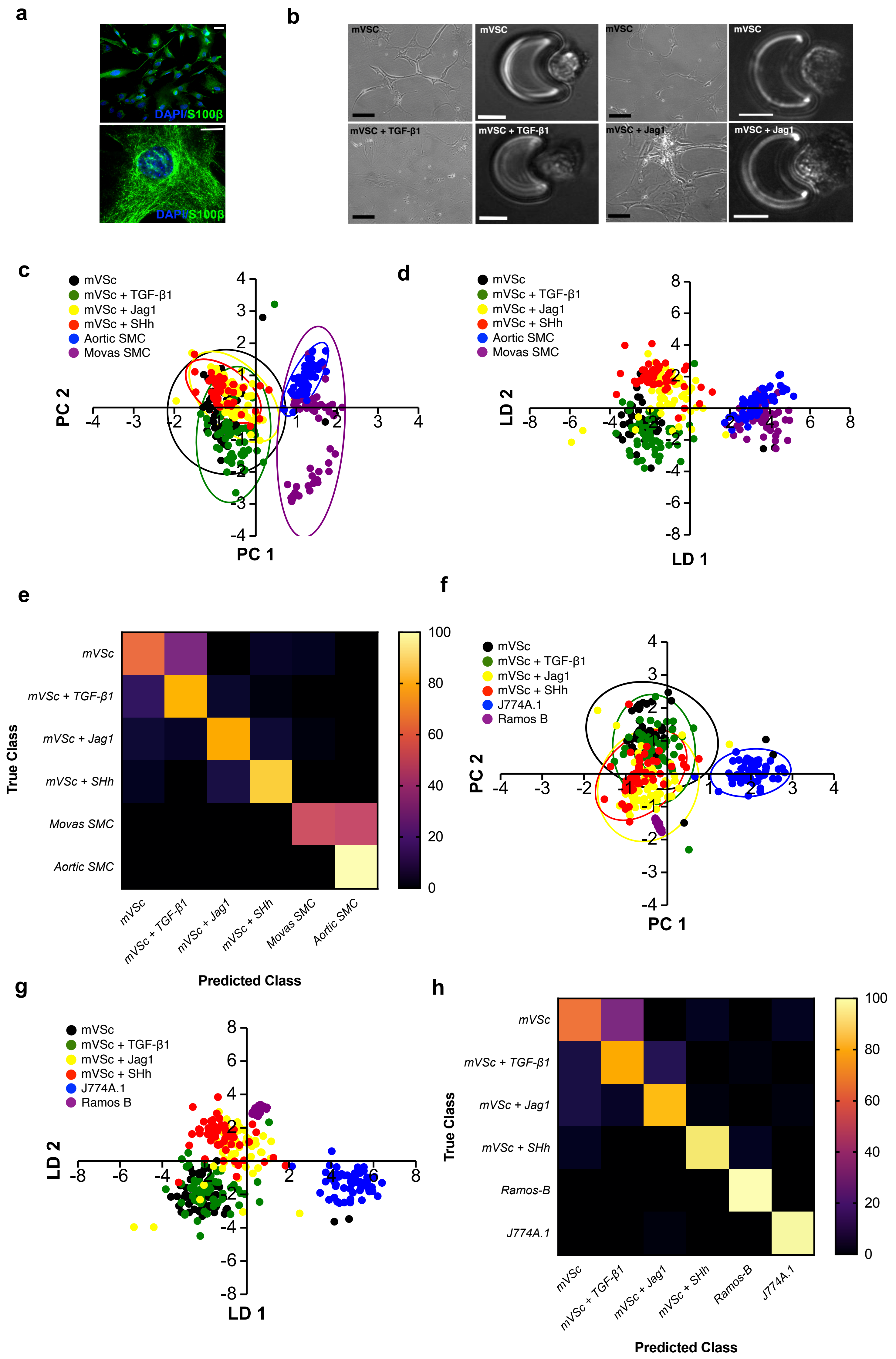
a
DAPI/S100β
DAPI/S100β
b
mVSC
mVSC
mVSC
mVSC
mVSC + TGF-β1
mVSC + TGF-β1
mVSC + Jag1
mVSC + Jag1
c
mVSc
mVSc + TGF-β1
mVSc + Jag1
mVSc + SHh
Aortic SMC
Movas SMC
PC 2
PC 1
d
mVSc
mVSc + TGF-β1
mVSc + Jag1
mVSc + SHh
Aortic SMC
Movas SMC
LD 2
LD 1
e
True Class
mVSc
mVSc + TGF-β1
mVSc + Jag1
mVSc + SHh
Movas SMC
Aortic SMC
Predicted Class
f
mVSc
mVSc + TGF-β1
mVSc + Jag1
mVSc + SHh
J774A.1
Ramos B
PC 2
PC 1
g
mVSc
mVSc + TGF-β1
mVSc + Jag1
mVSc + SHh
J774A.1
Ramos B
LD 2
LD 1
h
True Class
mVSc
mVSc + TGF-β1
mVSc + Jag1
mVSc + SHh
Ramos-B
J774A.1
Predicted Class

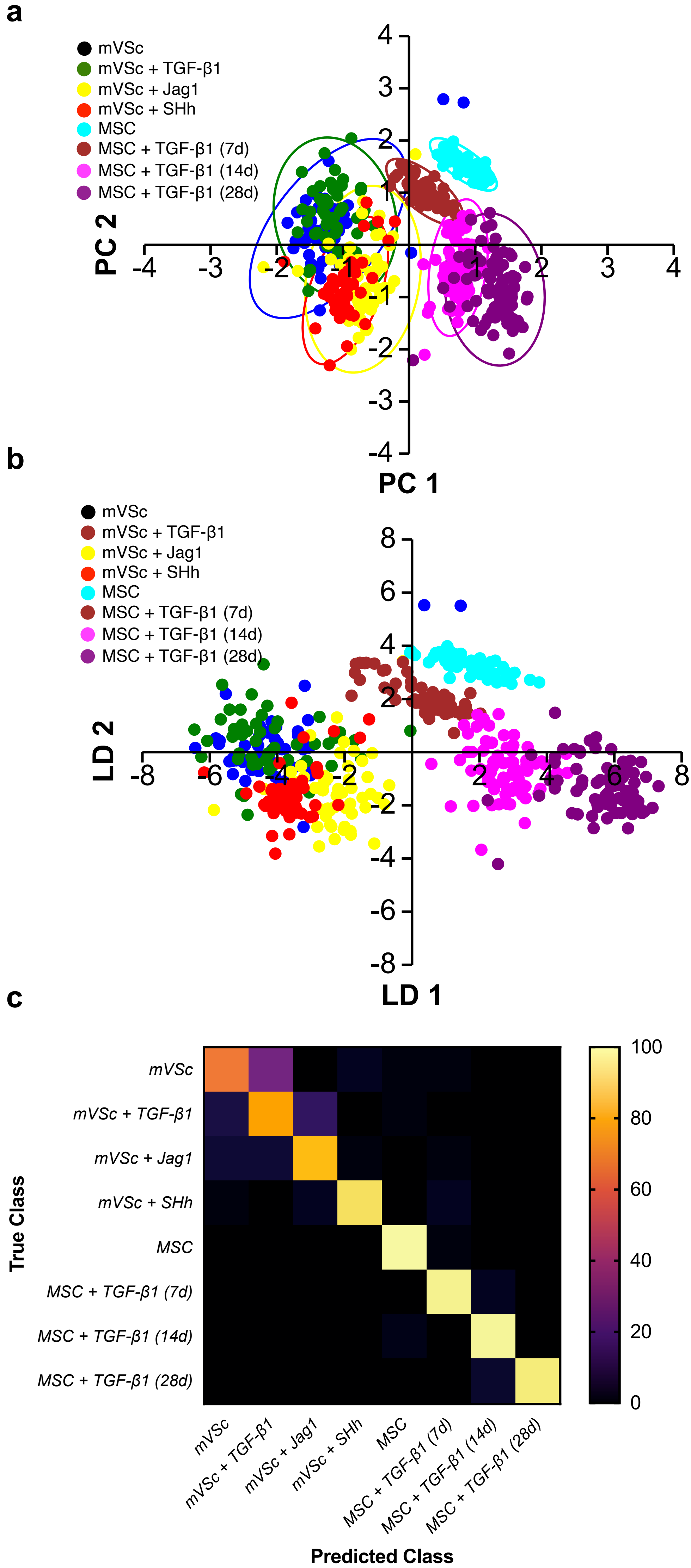

a
mVSc
mVSc + TGF-β1
mVSc + Jag1
mVSc + SHh
MSC
MSC + TGF-β1 (7d)
MSC + TGF-β1 (14d)
MSC + TGF-β1 (28d)
PC 2
PC 1
b
mVSc
mVSc + TGF-β1
mVSc + Jag1
mVSc + SHh
MSC
MSC + TGF-β1 (7d)
MSC + TGF-β1 (14d)
MSC + TGF-β1 (28d)
LD 2
LD 1
c
True Class
Predicted Class
mVSc
mVSc + TGF-β1
mVSc + Jag1
mVSc + SHh
MSC
MSC + TGF-β1 (7d)
MSC + TGF-β1 (14d)
MSC + TGF-β1 (28d)
100
80
60
40
20
0

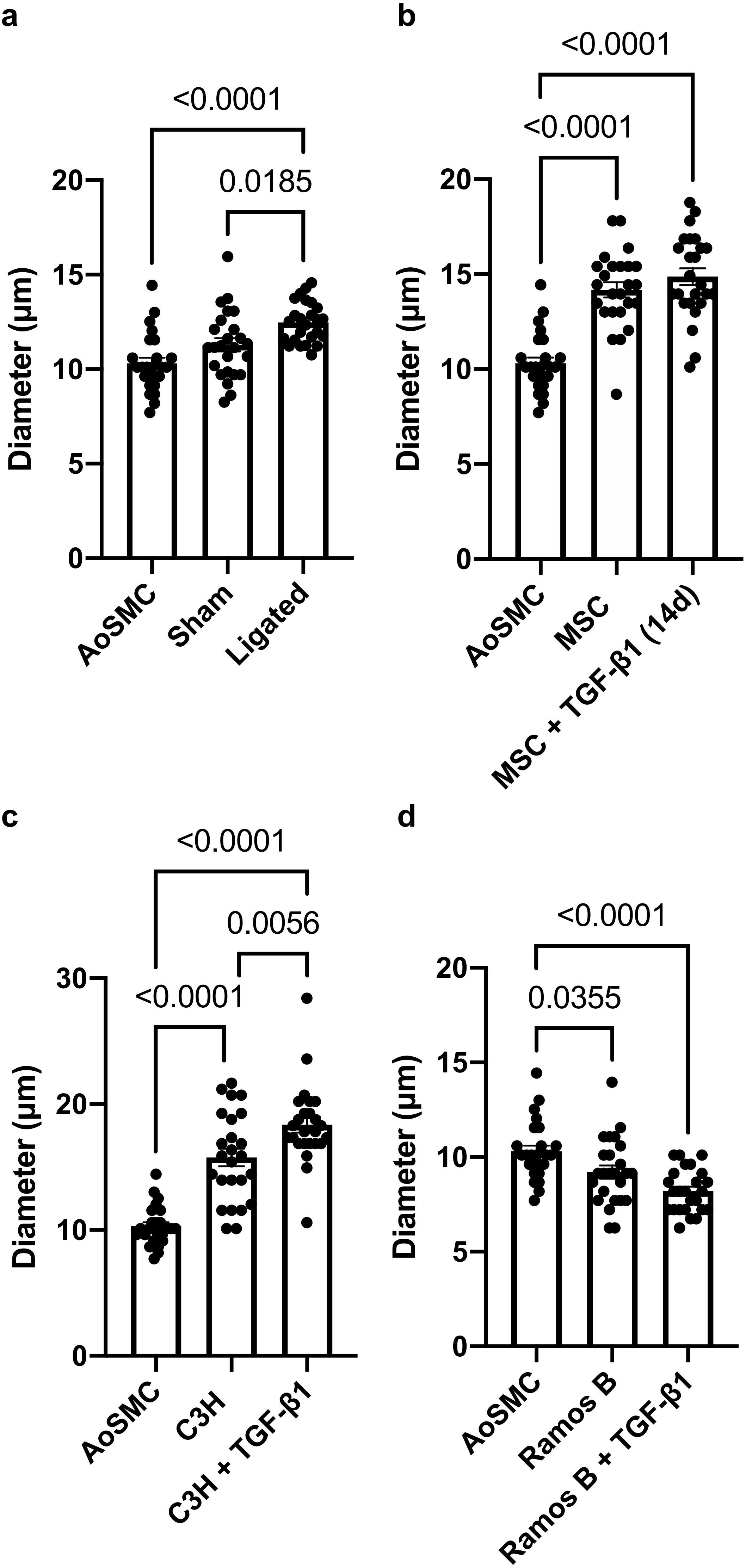
a
<0.0001
0.0185
Diameter (µm)
20
15
10
5
0
AoSMC
Sham
Ligated
b
<0.0001
<0.0001
Diameter (µm)
20
15
10
5
0
AoSMC
MSC
MSC + TGF-β1 (14d)
c
<0.0001
0.0056
<0.0001
Diameter (µm)
30
20
10
0
AoSMC
C3H
C3H + TGF-β1
d
<0.0001
0.0355
Diameter (µm)
20
15
10
5
0
AoSMC
Ramos B
Ramos B + TGF-β1

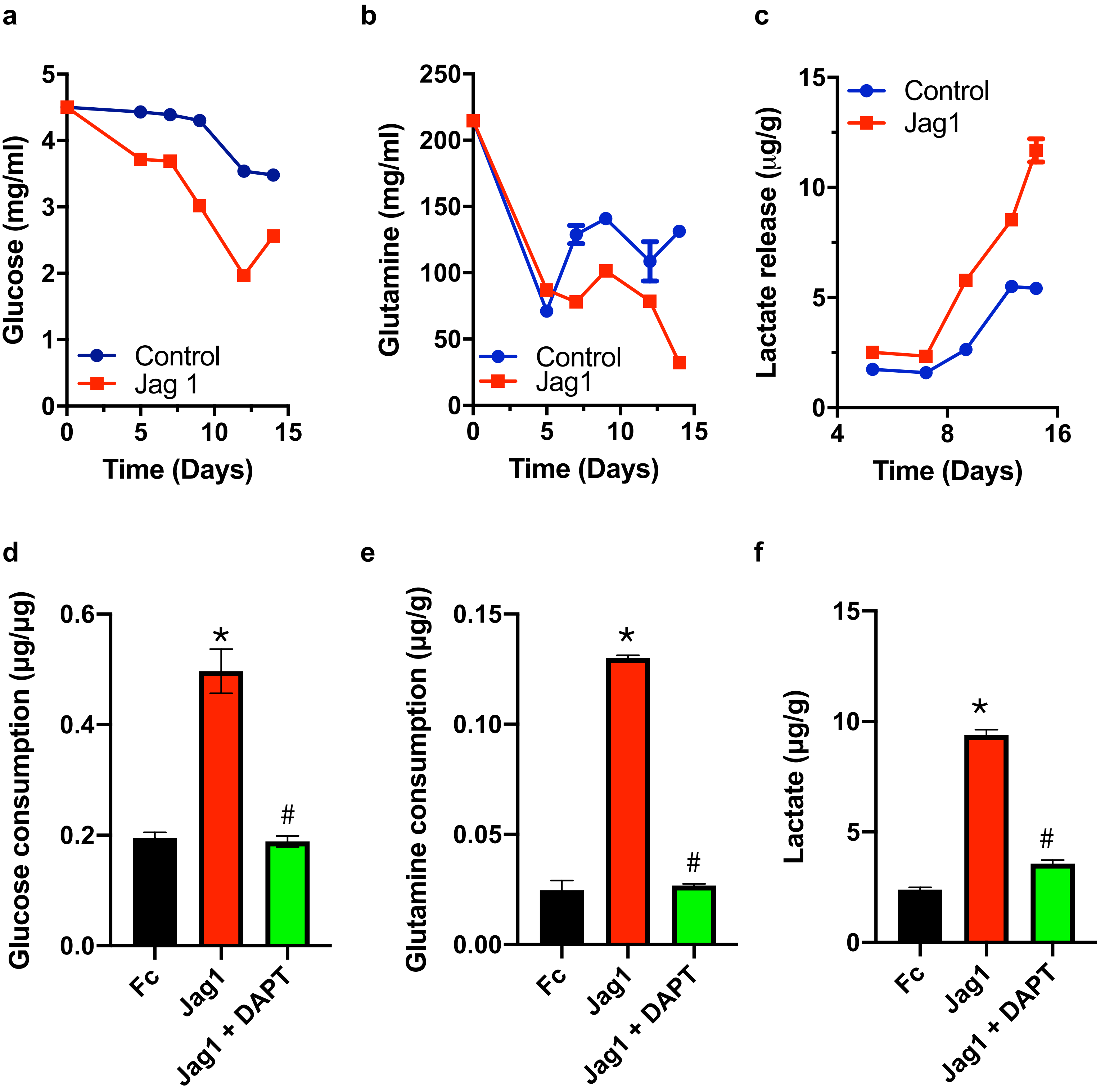
a
Glucose (mg/ml)
Time (Days)
Control
Jag 1
b
Glutamine (mg/ml)
Time (Days)
Control
Jag1
c
Lactate release (μg/g)
Time (Days)
Control
Jag1
d
Glucose consumption (μg/μg)
Fc
Jag1
Jag1 + DAPT
e
Glutamine consumption (μg/g)
Fc
Jag1
Jag1 + DAPT
f
Lactate (μg/g)
Fc
Jag1
Jag1 + DAPT

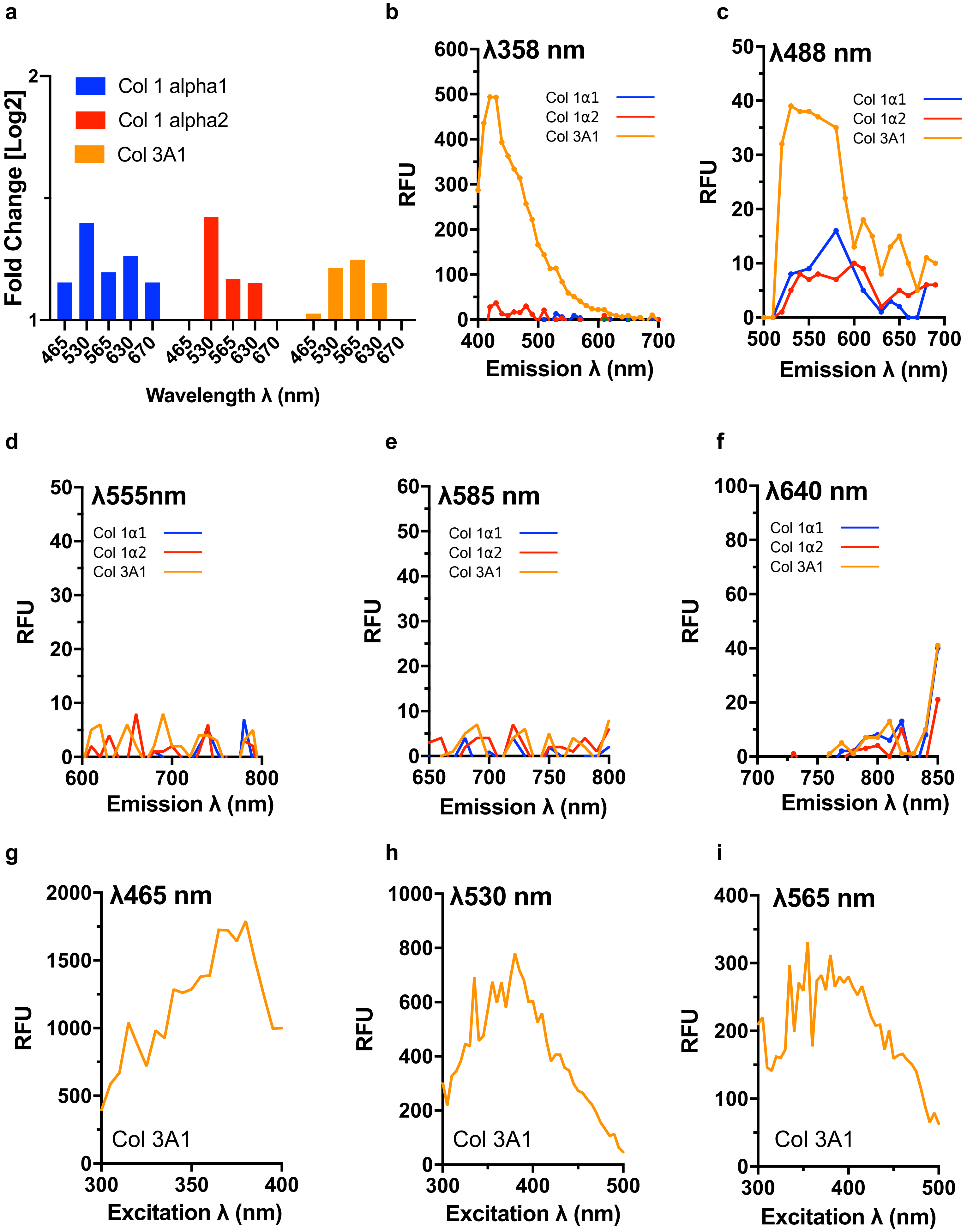
a
Fold Change [Log2]
2
1
Col 1 alpha1
Col 1 alpha2
Col 3A1
465 530 565 630 670 465 530 565 630 670 465 530 565 630 670
Wavelength λ (nm)
b
λ358 nm
600
500
400
300
200
100
0
RFU
Col 1α1
Col 1α2
Col 3A1
400 500 600 700
Emission λ (nm)
c
λ488 nm
50
40
30
20
10
0
RFU
Col 1α1
Col 1α2
Col 3A1
500 550 600 650 700
Emission λ (nm)
d
λ555nm
50
40
30
20
10
0
RFU
Col 1α1
Col 1α2
Col 3A1
600 700 800
Emission λ (nm)
e
λ585 nm
60
50
40
30
20
10
0
RFU
Col 1α1
Col 1α2
Col 3A1
650 700 750 800
Emission λ (nm)
f
λ640 nm
100
80
60
40
20
0
RFU
Col 1α1
Col 1α2
Col 3A1
700 750 800 850
Emission λ (nm)
g
λ465 nm
2000
1500
1000
500
0
RFU
Col 3A1
300 350 400
Excitation λ (nm)
h
λ530 nm
1000
800
600
400
200
0
RFU
Col 3A1
300 400 500
Excitation λ (nm)
i
λ565 nm
400
300
200
100
0
RFU
Col 3A1
300 400 500
Excitation λ (nm)
